# Influence of the TiO_2_ Precursor Phase on the Properties and Photoelectrooxidation Performance of Black TiO_2_-Impregnated Electrodes for Acetaminophen Degradation

**DOI:** 10.3390/molecules31091509

**Published:** 2026-05-01

**Authors:** Daniel Solarte-Ferro, John Betancourt, José A. Lara Ramos, Mario Millán-Franco, Jesús E. Diosa, Oscar A. Jaramillo-Quintero, Miguel Gracia-Pinilla, Fiderman Machuca-Martínez, Edgar Mosquera-Vargas

**Affiliations:** 1 Escuela de Ingeniería Química, Universidad del Valle, Santiago de Cali 760032, Colombia; solarte.daniel@correounivalle.edu.co (D.S.-F.); lara.jose@correounivalle.edu.co (J.A.L.R.); fiderman.machuca@correounivalle.edu.co (F.M.-M.); 2 Centro de Excelencia en Nuevos Materiales (CENM), Universidad del Valle, Santiago de Cali 760042, Colombia; betancourt.john@correounivalle.edu.co (J.B.); jesus.diosa@correounivalle.edu.co (J.E.D.); 3 Grupo de Transiciones de Fase y Materiales Funcionales, Departamento de Física, Facultad de Ciencias Naturales y Exactas, Universidad del Valle, Santiago de Cali 760042, Colombia; 4 Instituto de Física, Universidad Nacional Autónoma de México, Circuito de Investigación Científica s/n, Ciudad Universitaria, A.P. 20-364, Coyoacán C.P. 04510, Mexico; 5 Instituto de Energías Renovables, Universidad Nacional Autónoma de México, Privada Xochicalco s/n, Temixco C.P. 62580, Morelos, Mexico; oajaq@ier.unam.mx; 6 Facultad de Ciencias Físico Matemáticas, Universidad de Nuevo León, Universidad s/n, San Nicolás de los Garza C.P. 66455, Nuevo León, Mexico; miguel.graciapl@uanl.edu.mx; 7 Instituto de Materiales Avanzados para la Fabricación Sostenible, Tecnológico de Monterrey, Ave. Eugenio Garza Sada 2501, sur, Col. Tecnológico, Monterrey C.P. 64700, Nuevo León, Mexico

**Keywords:** black TiO_2_, dip-coating, pharmaceutical contaminants, photoelectrooxidation (PEO)

## Abstract

Black TiO_2_-impregnated electrodes were prepared via a modified dip-coating method, using six deposition layers to investigate the influence of the TiO_2_ precursor phase (anatase, rutile, and P25) on their structural and optical properties, as well as their photoelectrooxidation performance toward acetaminophen degradation. A reductive thermal treatment under a H_2_/Ar atmosphere successfully modified the band gap energy and promoted the formation of oxygen vacancies (Vo) and Ti^3+^ species, as evidenced by UV–Vis diffuse reflectance spectroscopy and photoluminescence analysis. Among the precursor phases, anatase exhibited the most significant band gap reduction, whereas rutile and P25 showed greater structural stability after the reduction process. Photoelectrochemical experiments revealed that the supporting electrolyte plays a dominant role in the degradation process, with significantly higher removal efficiencies observed in chloride medium (0.1 M NaCl) compared with sulfate medium (0.1 M Na_2_SO_4_) due to the formation of active chlorine species. Among the tested materials, rutile- and P25-derived electrodes showed the highest degradation efficiencies, reaching concentrations (C/C_0_) of 0.631 and 0.650, respectively. The results highlight the combined influence of precursor phase, defect structure, and electrolyte composition on the photoelectrooxidation behavior of black TiO_2_ electrodes and provide insights for the design of electrochemical systems for pharmaceutical contaminants removal.

## 1. Introduction

In recent years, increasing concern has arisen regarding the presence of pollutants in wastewater, driving the development of advanced treatment technologies capable of removing emerging contaminants. Highly sensitive analytical techniques have revealed the widespread occurrence of pharmaceutical compounds in aquatic environments, even at trace concentrations, where they may pose significant risks to ecosystems and human health [1,2,3,4]. Among these compounds, acetaminophen has received considerable attention due to its extensive global consumption and its continuous release through domestic and industrial effluents [5,6,7].

Acetaminophen is one of the pharmaceutical compounds most frequently detected in aquatic environments due to its widespread global consumption and continuous discharge from domestic wastewater and pharmaceutical industries. Conventional wastewater treatment plants are often inefficient at completely removing this compound due to its relatively stable aromatic structure and high-water solubility. Consequently, Acetaminophen is commonly used as a model contaminant to evaluate the performance of advanced oxidation processes in the removal of emerging pharmaceutical contaminants. Studying its degradation provides valuable information on the effectiveness of electrochemical and photoelectrochemical treatment technologies for water purification applications [8].

Conventional water treatment processes, such as coagulation, adsorption, and filtration, often exhibit limited efficiency at low contaminant concentrations, underscoring the need for alternative and more sustainable treatment strategies.

Advanced oxidation processes (AOPs) have emerged as promising alternatives due to their ability to generate highly reactive oxidative species capable of degrading a wide range of organic pollutants [8,9,10]. Photoelectrooxidation combines ultraviolet irradiation with the application of an electrical current, enhancing the formation of powerful oxidizing species that promote the degradation of recalcitrant contaminants [11,12]. The efficiency of this process is strongly dependent on the nature of the anode material, motivating the development of electrode materials with suitable electronic properties that favor charge transfer and the generation of reactive radicals [13].

Metal oxide semiconductors, including zinc oxide (ZnO) and tin oxide (SnO_2_), have been extensively investigated for environmental remediation due to their electronic structures and catalytic activity [14,15]. Among these materials, titanium dioxide (TiO_2_) has demonstrated particularly competitive performance owing to its chemical stability, low cost, and well-established photocatalytic properties [16,17,18,19]. More recently, black TiO_2_ has attracted increasing interest because of its narrowed band gap of 1.5 eV [20], defect-rich structure, and the presence of oxygen vacancies, which significantly enhance visible-light absorption and charge carrier dynamics [21,22,23,24]. Nevertheless, the photocatalytic and electrochemical performance of TiO_2_-based materials is strongly influenced by the crystalline precursor phase employed during synthesis.

The crystalline phase of TiO_2_ can significantly influence the formation and distribution of defect states generated during reductive treatments. Anatase and rutile possess distinct lattice structures and thermodynamic stability, which affect parameters such as lattice energy, hydrogen diffusion pathways, and the energy required for oxygen vacancy (*Vo*) formation. Rutile generally exhibits higher structural stability and electrical conductivity, while anatase tends to present a higher density of surface reactive sites and a more open crystal structure that may facilitate defect generation under certain conditions [25]. During hydrogen reduction, these intrinsic structural differences can lead to variations in the concentration and distribution of Ti^3+^ centers and oxygen vacancies (*Vo*), which are characteristic features of black TiO_2_ materials [20]. The presence of this disordered defect layer modifies the electronic structure of TiO_2_ by introducing localized states within the band gap, thereby improving charge carrier transport and influencing electrochemical properties such as charge transfer resistance and interfacial electron mobility. Consequently, the crystalline phase of the TiO_2_ precursor may play an important role in determining the final photoelectrochemical performance of black TiO_2_ electrodes. Therefore, systematic evaluation of precursor phases is essential to optimize the degradation performance toward pharmaceutical pollutants.

In addition to material composition, the deposition technique plays a critical role in determining electrode stability, surface morphology, and the availability of active sites. Several coating methods, such as spin coating, chemical bath deposition (CBD) and spray pyrolysis, have been explored to tailor TiO_2_ thin films and nanostructures, each offering distinct advantages depending on the targeted application. However, dip coating stands out as a cost-effective and versatile technique that enables the formation of uniform and reproducible films with controlled thickness. Key parameters, including solution viscosity and immersion and withdrawal velocities, can be readily adjusted to tailor coating properties [26,27,28,29,30,31]. Importantly, the TiO_2_ precursor phase also influences the formation of black TiO_2_ during reductive treatments and its subsequent behavior in photoelectrooxidation processes.

Based on these considerations, the working hypothesis of this study is that the crystalline phase of the TiO_2_ precursor influences the defect structure generated during hydrogen reduction and consequently affects the electronic properties and catalytic behavior of the resulting black TiO_2_ coatings. These structural and electronic differences may influence the interaction between the electrode surface and reactive species generated during electrochemical oxidation, ultimately impacting the efficiency of the photoelectrooxidation process for acetaminophen degradation.

In this work, the effectiveness of black TiO_2_-coated electrodes for the photoelectrooxidation degradation of acetaminophen is systematically evaluated in chloride and sulfate media. The influence of the TiO_2_ precursor phase (anatase, rutile, P25, and anatase–rutile mixtures) on the synthesis of black TiO_2_ via hydrogen thermal treatment is investigated, along with its deposition onto titanium substrates using the dip-coating method. Comprehensive structural, morphological, and optical characterization is conducted to elucidate the physicochemical properties of the prepared materials. These results were correlated with the degradation performance to establish structure-activity relationships governing the photoelectrochemical removal of pharmaceutical contaminants.

## 2. Materials and Methods

### 2.1. Materials

All reagents used in this study were of analytical grade and were employed without further purification. For the synthesis of black TiO_2_, three titanium dioxide precursors were used: commercial AEROXIDE^®^ TiO_2_ P25 powder (Evonik Corporation, Parsippany, NJ, USA, Norh America, CAS-No. 13463-67-7, ≥99.5% purity), commercial anatase TiO_2_ (Sigma-Aldrich, Darmstadt, Germany, CAS-No 1317-70-0, <25 nm particle size, 99.7% purity), and commercial rutile TiO_2_ powder (Sigma-Aldrich, CAS-No 1317-80-2, <5 µm particle size, 99.9% purity). Commercial urea (CH_4_N_2_O, Merck, Darmstadt, Germany, CAS-No 57-13-6, analytical standar) was used as a co-doping agent in the solid-state treatment.

Reductive thermal treatments were conducted under two different atmospheres: a hydrogen–argon gas mixture (5.4% H_2_ and 94.6% Ar, supplied by Messer Colombia, Cota, Colombia) and nitrogen gas (N_2_, 96% purity, supplied by Messer Colombia). Triton X-100 (C_8_H_17_C_6_H_4_(OCH_2_CH_2_)_7_nOH, Sigma-Aldrich, CAS-No 92046-34-9) was employed as a surfactant during electrode impregnation. Acetaminophen (C_8_H_9_NO_2_) was used as a model pharmaceutical compound for degradation experiments. Sodium chloride (NaCl, Sigma-Aldrich, CAS-No 54024-22-5, ≥99% purity) and sodium sulfate (Na_2_SO_4_, Fisher Chemical^TM^, Waltham, MA, USA, CAS-No 7757-82-6, 99.9% purity) were used to prepare electrolyte solutions.

### 2.2. Sample Preparation

Four TiO_2_ precursor materials were used for the synthesis: P25 powder, anatase nanopowder, and rutile powder. An additional sample was prepared via a solid-state chemical reaction by mixing 20% rutile and 80% anatase phases. Furthermore, a co-doping process was carried out by incorporating urea into each precursor through solid-state mixing at a concentration of 20 wt%. All prepared samples and their corresponding compositions are summarized in Table 1.

### 2.3. Black TiO_2_ Synthesis

**Scheme 1:**
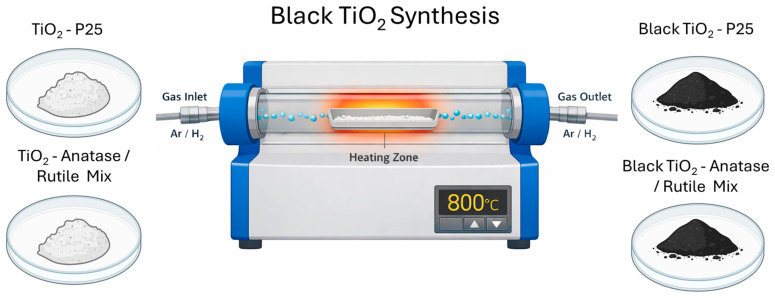
Experimental thermal reduction treatment of the samples to obtain black TiO_2_.

For each TiO_2_ precursor phase, a thermal reduction treatment under a hydrogen-containing atmosphere was employed, following previously reported procedures with appropriate modifications [22,32,33,34,35,36]. The samples were placed in ceramic crucibles and introduced into a tubular furnace (Scheme 1). Prior to thermal treatment, the system was purged with nitrogen (N_2_) for 15 min to remove residual air. Subsequently, the samples were calcined under a hydrogen–argon atmosphere (H_2_/Ar) at a total gas flow rate of 10 sccm. The treatment was carried out at 800 °C for 8 h, using heating and cooling rates of 10 °C min^−1^. After cooling to room temperature, the obtained black TiO_2_ samples were collected, stored under ambient conditions, and labeled using the same identification scheme as the untreated samples, with the addition of the prefix “T”.

### 2.4. Characterization Techniques

The structural, morphological, and optical characterization of pure, N,C solid-state co-doped, and thermally treated TiO_2_ samples was carried out using a combination of complementary techniques. Scanning electron microscopy coupled with energy-dispersive X-ray spectroscopy (Thermo Fisher Scientific Phenom Pro X Destop SEM/EDS, Waltham, MA, USA) was employed to examine surface morphology and elemental composition, using an accelerating voltage of 15 kV. UV–Vis diffuse reflectance spectra (UV–Vis DRS) were recorded at room temperature using a UV–Vis/NIR spectrophotometer (V-770, Jasco Inc.) over a wavelength range of 200–1000 nm [24,34,35]. The optical band gap energies were estimated using the Kubelka–Munk function.

Photoluminescence (PL) measurements were performed at room temperature with a spectrofluorometer (FP-8250, Jasco, Tokyo, Japan) in the wavelength range of 300–750 nm [34,35]. Fourier-transform infrared (FTIR) spectra were collected using an FTIR spectrophotometer (FT/IR-6800, Jasco) in the range of 4000–350 cm^−1^ [32,35]. Raman spectroscopy was conducted using a Raman spectrometer (RMS-4500, Jasco) equipped with a 532 nm laser, and spectra were recorded in the range of 50–4000 cm^−1^ [35].

### 2.5. Electrode Coating Procedure

Black TiO_2_ nanoparticles were dispersed in Type I deionized water at a concentration of 15% (*w*/*v*), with the addition of one drop of Triton X-100 as a surfactant. Each suspension was sonicated for 1 h at 60 kHz to obtain homogeneous particle dispersion.

Titanium electrodes with dimensions of 2.0 × 2.5 cm and a thickness of 5 mm were coated using the dip-coating technique [26,27,28,29]. The electrodes were immersed in the black TiO_2_ suspension at a controlled withdrawal and immersion rate of 10 cm min^−1^, held in the solution for 1 min, and then withdrawn. Immediately after coating, the electrodes were placed in a furnace and heated at a ramp rate of 5 °C min^−1^ up to 150 °C, where they were maintained for 15 min to promote partial adhesion of the coating.

Subsequently, the electrodes were quenched in Type I deionized water to remove loosely attached particles and returned to the furnace for an additional 15 min. This sequence constituted one coating cycle and was repeated until six coating layers were achieved. The deposited mass on each electrode was determined gravimetrically by calculating the difference between the mass of the coated electrode and that of the uncoated substrate, as expressed in Equation $m_{d}=m_{f}-m_{i}$, where $m_{d}$ is the deposited mass, $m_{f}$ is the mass of the electrode after coating, and $m_{i}$ is the initial mass of the uncoated electrode.

### 2.6. Photoelectrooxidation of Acetaminophen

Acetaminophen degradation experiments were carried out in a two-electrode electrochemical cell with a total working volume of 100 mL, using either 0.1 M NaCl (chloride medium) or 0.1 M Na_2_SO_4_ (sulfate medium) as the supporting electrolyte [36,37]. Black TiO_2_-coated titanium electrodes with a geometric area of 5 cm^2^ were used as both the anode and cathode during the degradation tests, which were conducted for 90 min.

A DC power supply was connected in series with a multiparameter meter to enable real-time monitoring and control of the applied current. The current was fixed at 12.5 mA, corresponding to a current density of 2.5 mA cm^−2^. Continuous magnetic stirring at 500 rpm was applied throughout the experiments to ensure adequate mass transport. The electrochemical cell was placed inside a homemade photoreactor equipped with a UV–visible lamp to irradiate the system during operation. The emission spectrum of the lamp exhibited main peaks at 436, 547, 405, 579, 399, and 313 nm.

Acetaminophen concentration was quantified using a Shimadzu LC-2010A HT Liquid Chromatograph equipped with an ODS2-SL5 column (5 µm particle size, 4.6 × 250 mm). The mobile phase consisted of a mixture of acetonitrile (Merk, CAS-No 75-05-8, gradient grade, suitable for HPLC) and Type I water in a 40:60 (*v*/*v*) ratio. The flow rate was set to 0.5 mL min^−1^, and detection was performed using a UV detector at 243 nm [36,38,39].

## 3. Results and Discussion

### 3.1. SEM and EDS Analysis

**Figure 1:**
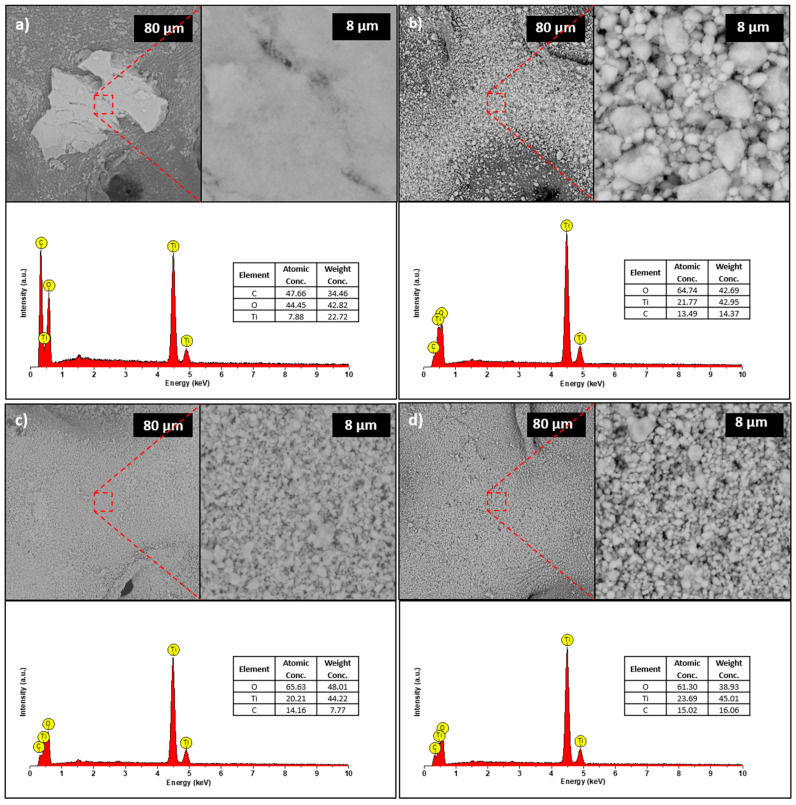
Morphological and compositional analysis of TiO_2_ samples (P25, R, A, M). (**a**) pristine commercial samples; (**b**) N, C co-doped samples; (**c**) thermally treated commercial samples; and (**d**) thermally treated N, C co-doped samples.

**Figure 2:**
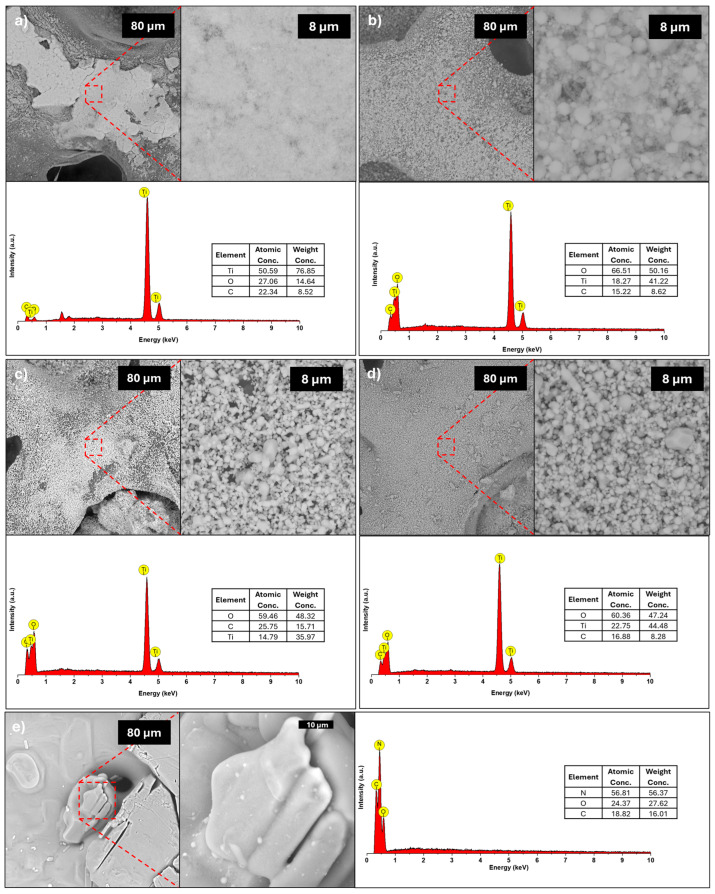
Morphological and compositional analysis of N, C co-doped TiO_2_ samples: (**a**) N, C pristine commercial (N,C P25); (**b**) N, C Rutile; (**c**) N, C Anatase, (**d**) N, C Mixture, and (**e**) Urea.

Figure 1a–d and Figure 2a–d present SEM micrographs of the TiO_2_ precursor samples and the N,C solid-state co-doped TiO_2_ materials obtained using urea. The microstructural analysis indicates that the pristine precursors exhibit predominantly well-defined, quasi-spherical particle morphologies. An exception is observed for the P25 sample, which displays a more irregular and heterogeneous morphology, a characteristic commonly reported for this commercial mixed-phase powder [40,41].

Following the N,C solid-state co-doping treatment, all samples showed a slight increase in particle agglomeration. Nevertheless, the overall particle shape was largely preserved for the anatase, rutile, and mixed-phase samples, suggesting that the co-doping process did not induce significant morphological alterations in the TiO_2_ particles. In the case of P25, the irregular morphology remained unchanged, further supporting the conclusion that the solid-state treatment primarily affects surface chemistry rather than particle structure.

Energy-dispersive X-ray spectroscopy (EDS) was employed to evaluate the elemental composition and atomic ratios of the samples. Prior to co-doping, the P25 sample exhibited a comparatively higher molar content of carbon and oxygen than the anatase, rutile, and mixed-phase samples, which may be attributed to its industrial manufacturing process or the presence of residual organic species [42]. After the N,C co-doping treatment, an increase in the atomic concentration of carbon was observed across all samples, confirming the successful incorporation of carbon-containing species on the particle surfaces. No detectable nitrogen signal was observed in the EDS spectra of the co-doped materials, likely due to the low dopant concentration. For all precursor phases, oxygen remained the predominant element both before and after the co-doping process, consistent with the TiO_2_ matrix [43].

### 3.2. Vibrational Spectroscopy

**Figure 3:**
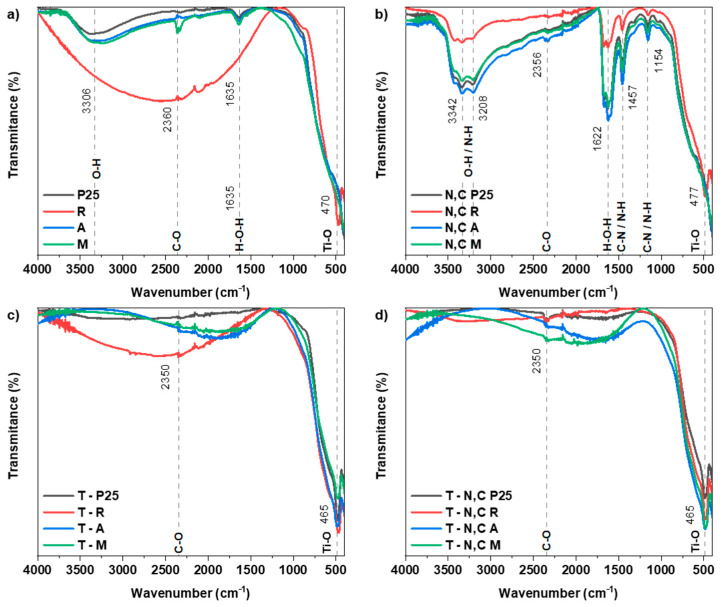
FTIR of TiO_2_ samples (P25, R, A, M). (**a**) pristine commercial samples; (**b**) N, C co-doped samples; (**c**) thermally treated commercial samples; and (**d**) thermally treated N, C co-doped samples.

The FTIR spectra shown in Figure 3a–d displays the characteristic vibrational features of TiO_2_-based materials. Bands observed in the range of 465–477 cm^−1^ is assigned to Ti–O and Ti–O–Ti stretching vibrations, confirming the preservation of the TiO_2_ lattice structure [44,45,46]. Broad absorption bands centered between 3200 and 3400 cm^−1^, together with a signal near 1630 cm^−1^, are attributed to O–H stretching and bending modes, respectively, indicating the presence of surface-adsorbed water molecules and hydroxyl groups [47].

For the N,C co-doped samples, additional bands emerge at approximately 1154, 1457, and 1622 cm^−1^. These features can be associated with vibrational modes related to C–N, C–O, and N–H bonds, providing spectroscopic evidence for the incorporation of carbon- and nitrogen-containing species during the solid-state co-doping process. A weak absorption band detected in the 2320–2360 cm^−1^ region is attributed to the asymmetric stretching vibration of adsorbed CO_2_ species [48].

Following the thermal reduction treatment, the vibrational bands associated with urea-derived organic functionalities (C–N, N–H, and C=O) largely disappear from the spectra [49,50,51,52,53,54,55]. This observation indicates that the organic dopant species decomposed during thermal processing, leaving the TiO_2_ lattice vibrations as the dominant spectral features. Residual contributions from surface hydroxyl groups and adsorbed CO_2_ remain detectable; however, the overall spectral profile confirms that the structural integrity of the TiO_2_ framework was maintained after thermal treatment.

**Figure 4:**
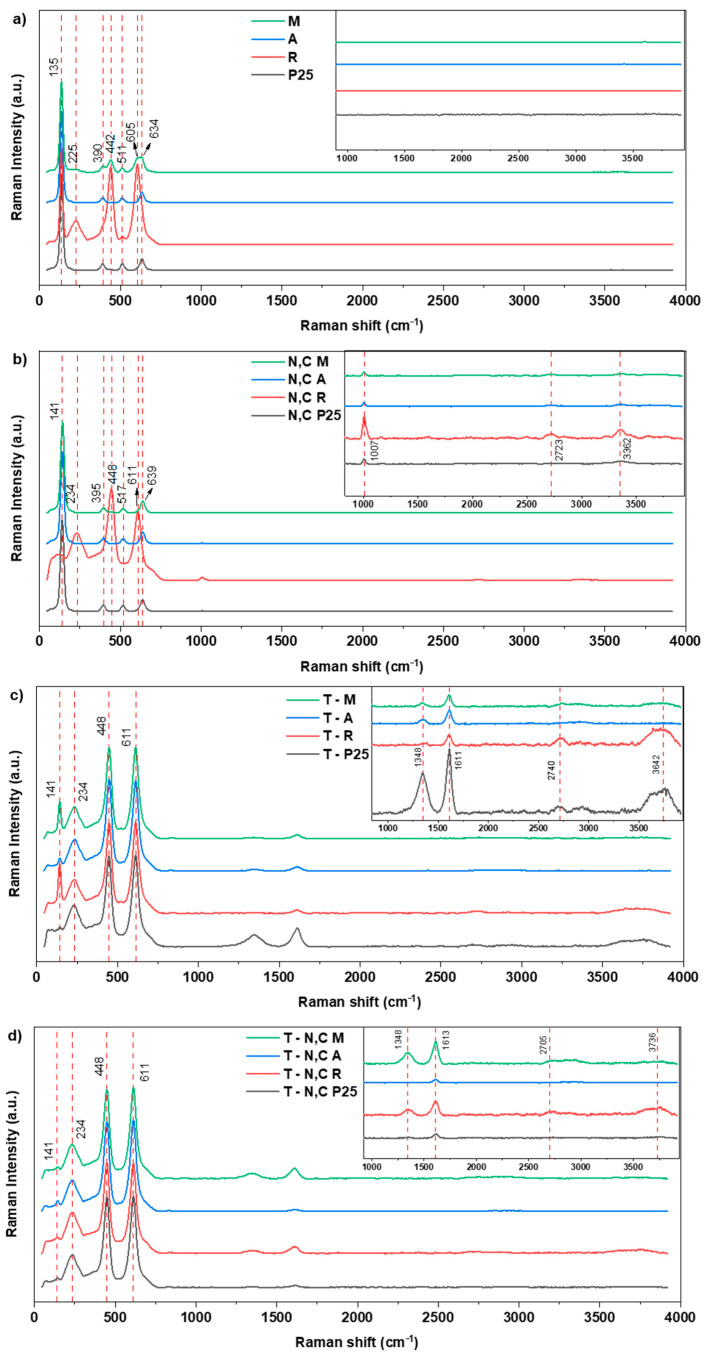
Raman spectra of TiO_2_ samples (P25, R, A, M). (**a**) commercial samples; (**b**) N,C co-doped samples; (**c**) thermal treated commercial samples; (**d**) thermal treated N,C co-doped samples.

The Raman spectra presented in Figure 4a–d exhibit the characteristic vibrational fingerprints of TiO_2_, with bands located at approximately 141, 189, 225, 390, 448, 511, and 634 cm^−1^. These peaks are assigned to the characteristic E_g_, B_1g_, and A_1g_ vibrational modes of TiO_2_ polymorphs. The mixed-phase samples (R, A, and M) display features attributable to both rutile and anatase phases, confirming their composite crystalline nature [42,43].

Raman spectroscopy was used as the primary technique to identify the crystalline phases of the TiO_2_ coatings. This method provides detailed information about the vibrational modes associated with the anatase and rutile polymorphs and is particularly suitable for the characterization of thin films and nanostructured materials where the amount of deposited material may be limited. The observed Raman bands correspond well with the characteristic modes reported for anatase and rutile TiO_2_, indicating that the precursor phases are preserved after the hydrogen reduction treatment. Although X-ray diffraction (XRD) is commonly employed for phase identification in TiO_2_ materials, Raman spectroscopy has also been widely used as a reliable tool for distinguishing TiO_2_ polymorphs and detecting local structural variations in photocatalytic systems.

For the N,C co-doped materials, additional Raman features are observed alongside the Ti–O lattice vibrations, particularly in the regions of 1004–1010 cm^−1^ and 2700–3360 cm^−1^ [49,50]. These bands are attributed to residual organic moieties or vibrational modes associated with nitrogen- and carbon-containing species derived from the urea precursor, indicating partial retention of dopant-related functional groups after the solid-state treatment [51].

Following the thermal reduction process, the Raman spectra of the N,C co-doped samples show a pronounced attenuation or complete disappearance of bands associated with urea-derived species. This effect is especially evident for the T-N, C A sample, which exhibits a clean spectral profile dominated by the characteristic TiO_2_ vibrational modes. These results indicate that the applied thermal protocol effectively removes residual organic fragments while preserving the crystallinity and structural integrity of the TiO_2_ lattice [52].

The persistence of the TiO_2_ Raman modes across all thermally treated samples further demonstrates that the H_2_-based thermal process primarily alters the electronic structure, as corroborated by the observed band gap energy in the UV–vis analysis, without inducing significant changes in the crystalline framework [51]. This suggests that the generated defects are predominantly surface-localized, promoting the formation of active sites while maintaining the structural stability required for electrode applications.

It is important to note that thermal treatments at elevated temperatures can induce structural transformations in TiO_2_ polymorphs. In particular, anatase is known to gradually transform into rutile when heated in the range of approximately 700–900 °C, depending on particle size, atmosphere, and synthesis conditions. However, in the present study the reductive thermal treatment was performed under controlled conditions that were not expected to promote significant phase transformation. The Raman spectra confirms that the characteristic vibrational modes of each TiO_2_ polymorph remain preserved after treatment, indicating that the reduction process primarily introduces defect states such as oxygen vacancies without altering the crystalline framework. Similar observations have been reported in previous studies investigating TiO_2_ morphology and defect-related photocatalytic activity [28].

Although the spectroscopic signals of urea-derived functional groups disappear after thermal treatment, the initial co-doping step may still influence the final material properties. The presence of urea during the solid-state process can modify the local chemical environment and promote the formation of oxygen vacancies and Ti^3+^ species during subsequent hydrogen reduction. Therefore, its role is likely indirect, acting as a transient agent that directs the structure or promotes defects, rather than a permanent dopant in the final black TiO_2_ structure.

### 3.3. Optical Properties of the Samples

**Figure 5:**
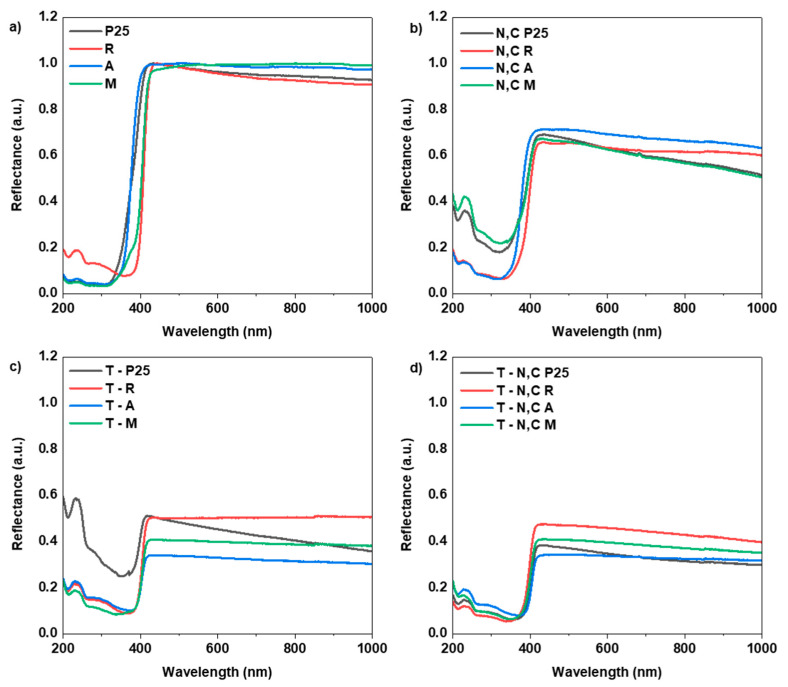
UV-vis DRS of TiO_2_ samples (P25, R, A, M). (**a**) pristine commercial samples; (**b**) N, C co-doped samples; (**c**) thermal treated commercial samples; (**d**) thermal treated N, C co-doped samples.

Figure 5a–d displays the UV–Vis diffuse reflectance spectra (UV–Vis DRS) of pristine, urea co-doped, and thermally treated TiO_2_ samples. UV–Vis DRS was employed to evaluate the optical band gap energies using the Kubelka–Munk function, *F*(*R*), which relates diffuse reflectance to absorption, as expressed in Equation (1) [41,46]:

(1) $$F\left(R\right)=\frac{{\left(1-R\right)}^{2}}{2R}$$

**Figure 6:**
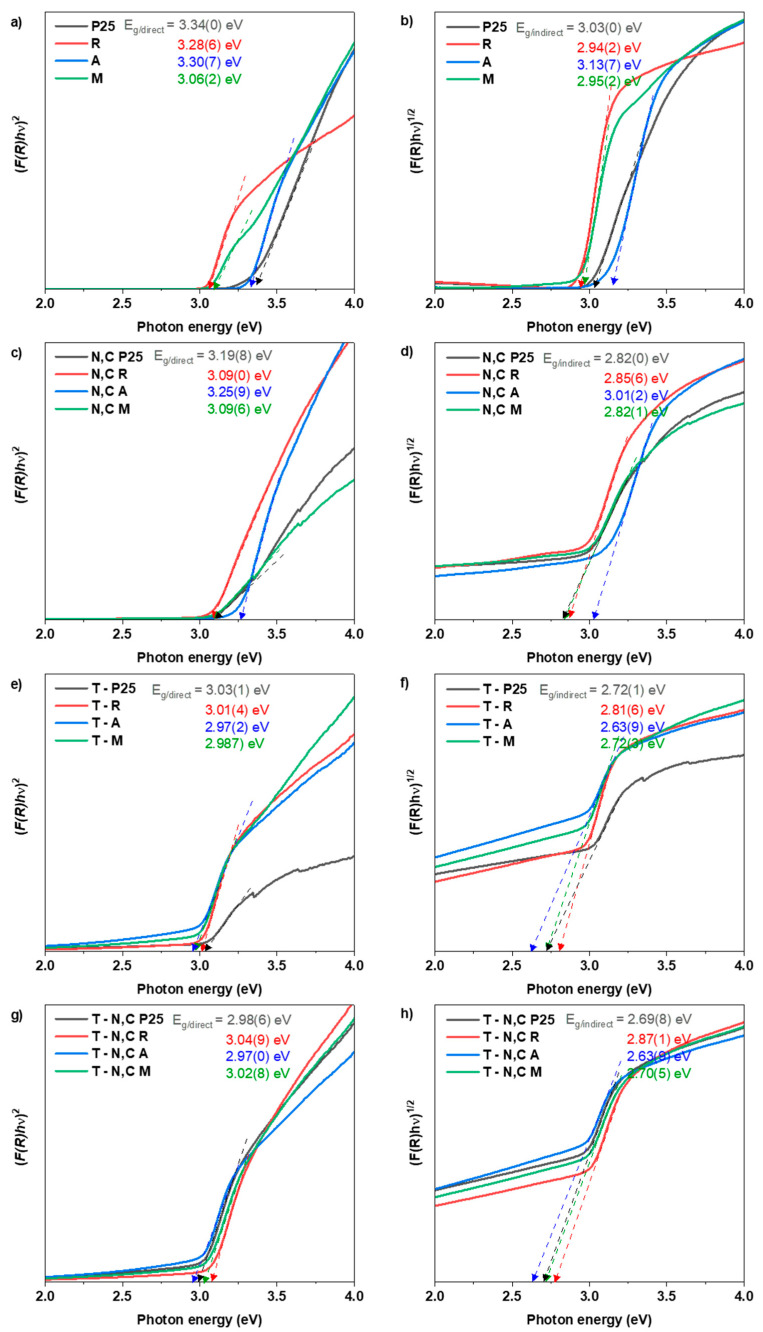
Kubelka-Munk plots and band gap energy estimation of TiO_2_ samples for direct and indirect transitions. (**a**,**b**) pristine commercial samples; (**c**,**d**) N, C co-doped samples; (**e**,**f**) thermally treated commercial samples; and (**g**,**h**) thermally treated N, C co-doped samples.

The band gap energies were determined by plotting $[F(R)hv{]}^{1/2}$ as a function of photon energy, hv, where h is Planck’s constant and v is the photon frequency, to estimate the indirect band gap (Figure 6) [43,45,46,47]. In the linear region of the plot, the extrapolation of the fitted line to the energy axis yielded the indirect band gap value according to $E_{g}=-b/m$, where b and m represent the intercept and slope of the linear fit, respectively. The associated uncertainty was obtained by error propagation from the standard errors of the fitting parameters. An analogous procedure was applied to determine the direct band gap energies by plotting $[F(R)hv{]}^{2}$, as shown in Figure 6. The calculated indirect and direct band gap values are summarized in Table 2.

As shown in Figure 5, the UV–vis DRS spectra of the pristine and N,C co-doped TiO_2_ samples subjected to thermal treatment exhibit a noticeable decrease in reflectance intensity compared to their untreated counterparts. This behavior indicates enhanced light absorption, which can be attributed to the formation of defect-related states and dopant-induced energy levels within the band structure [48,49]. Pristine TiO_2_ samples display typical band gap values of approximately 3.3 eV (direct) and 3.0 eV (indirect), consistent with reported values for anatase- and rutile-based TiO_2_ materials. A slight reduction in band gap energy is observed for the N,C co-doped samples, suggesting the introduction of dopant-related states that modify the electronic structure.

Following thermal treatment under a reducing H_2_/Ar atmosphere, all samples exhibit a pronounced narrowing of both direct and indirect band gaps. This effect is particularly evident for the treated anatase sample, which shows band gap values of 2.97 eV (direct) and 2.64 eV (indirect). Such behavior is indicative of the formation of oxygen vacancies and Ti^3+^ centers within the TiO_2_ lattice [52,53,54,55]. The combined effect of dopant incorporation and reductive thermal treatment promotes the generation of localized mid-gap states, extending optical absorption into the visible region and potentially enhancing the photoactivity of the material.

**Figure 7:**
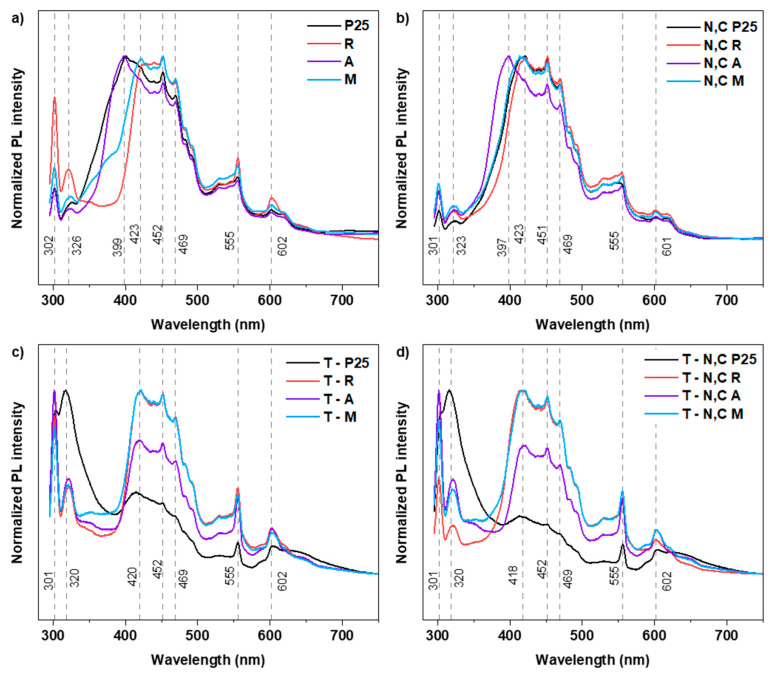
PL spectra of TiO_2_ samples (P25, R, A, and M) illustrating the effects of N, C co-doping and thermal treatment: (**a**) pristine commercial samples; (**b**) N, C co-doped samples; (**c**) thermally treated pristine samples; and (**d**) thermally treated N, C co-doped samples.

Figure 7 presents the photoluminescence (PL) spectra of all TiO_2_ samples. The PL results provide direct evidence of defect states previously inferred from the UV–vis DRS analysis. As the spectra were normalized, an intensity ratio analysis was performed relative to a reference emission to enable accurate comparison of variations among the different samples. All materials exhibit characteristic emission bands that are consistent with previously reported PL features of TiO_2_-based systems [52,55].

The pristine TiO_2_ samples show dominant emission in the central spectral region (399–469 nm), which is commonly associated with oxygen vacancies and sub-band-gap defect states related to Ti^3+^ mid-gap levels. Upon introduction of N, C dopants, a redistribution of the PL intensity is observed. Specifically, an enhancement of the central emission region occurs across all phases, indicating the formation of additional defect sites, such as oxygen vacancies, induced by the co-doping process [54]. In the rutile sample, the dominant emission peak is significantly attenuated, suggesting that dopant incorporation introduces non-radiative recombination pathways that alter charge carrier dynamics. Similarly, the P25 sample exhibits the suppression of the emission near 397 nm, which may indicate improved charge separation or modification of electronic states at the anatase–rutile interface.

After thermal treatment under reducing conditions, all samples exhibit pronounced spectral changes. The P25 sample shows the most significant modification, characterized by the emergence of a strong emission peak near 320 nm, which dominates the spectrum and suppresses the central emission region. This feature is attributed to defect states associated with oxygen vacancies, reduced Ti sites, or structural modifications induced by the hydrogen-containing atmosphere [48]. In contrast, the anatase and mixed-phase samples display PL profiles similar to that of the initial rutile sample, suggesting that rutile is less sensitive to defect redistribution, likely due to its higher structural stability and the absence of phase transformation during thermal treatment.

Finally, the co-doped and thermally treated samples follow trends similar to those observed for the untreated thermally reduced materials, confirming that the reducing atmosphere plays a dominant role in defect formation. Notably, rutile exhibits a pronounced decrease in emission intensity at both 302 and 320 nm, suggesting that the presence of dopant species contributes to the passivation or redistribution of deep-level defects generated during the reduction process [54,55].

### 3.4. Deposition of Black TiO_2_ on Electrodes

The deposition of black TiO_2_ onto titanium sheet substrates using a controlled dip-coating technique was successfully achieved. A six-layer configuration was selected to avoid excessive material accumulation while ensuring the mechanical stability of the electrodes. The quenching steps proved effective for removing loosely attached particles and promoting tighter packing of the coating on the electrode surface. As a result, the final films exhibited good uniformity and adhesion.

Table 3 summarizes the mass deposited on both the anode and cathode for each precursor phase. The black TiO_2_ anatase coating produced the highest deposited mass, reaching 0.0041 g on the anode and 0.0043 g on the cathode, which suggests stronger particle substrate interactions and improved dispersion during the coating process. In contrast, the rutile-coated electrodes exhibited the lowest deposited mass, likely due to the larger particle size and lower surface area of rutile, which may limit layer adhesion during dip coating.

### 3.5. Photoelectrooxidation for Acetaminophen Degradation

Photoelectrooxidation experiments were conducted to evaluate the degradation performance of acetaminophen on platinum electrodes coated with different precursor phases of black TiO_2_ (uncoated electrode used as reference). In these experiments, two supporting electrolytes were employed: Na_2_SO_4_ and NaCl, both at a concentration of 0.1 M and under identical conditions of current density (2.5 mA cm^−2^), solution volume (100 mL), and operating time (90 min).

**Figure 8:**
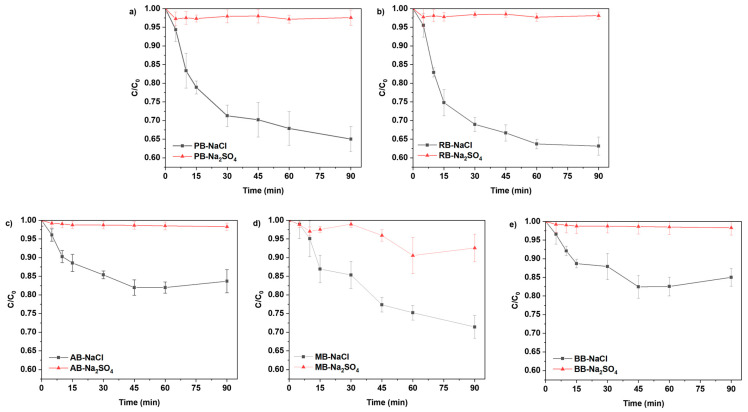
Acetaminophen degradation profiles in 0.1 M NaCl and 0.1 M Na_2_SO_4_ electrolytes for (**a**) black P25, (**b**) black rutile, (**c**) black anatase, (**d**) black mixed phase, and (**e**) the non-coated electrode.

The tests performed with Na_2_SO_4_ as the supporting electrolyte did not reach 10% degradation. In particular, the mixed-phase coating (ID: M) reached a final C/C_0_ value of 0.926 (see Figure 8) [36]. In contrast, the experiments conducted with NaCl as the electrolyte exhibited degradation rates between 15% and ~37%. Specifically, the highest degradation percentages were obtained with electrodes coated with rutile and P25 precursor phases, which reached minimum C/C_0_ values of 0.631 and 0.650, respectively.

Compared with the uncoated electrode (C/C_0_ = 0.850), the improvement achieved through the coating process can be considered moderate. Although the coated electrodes consistently exhibited superior performance, the degradation efficiency did not increase proportionally with prolonged reaction times under the evaluated conditions. This behavior suggests a possible transition towards a mass-transfer-controlled regime or the occurrence of parasitic competitive reactions.

The superior band gap modification observed for anatase-derived black TiO_2_ does not directly translate into higher degradation efficiency, indicating that optical absorption is not the sole determining factor. While band gap reduction enhances light harvesting, rutile and P25 phases typically exhibit higher electrical conductivity and improved charge carrier mobility, which favor interfacial electron transfer during electrochemical reactions. Additionally, mixed-phase systems, such as P25, can promote charge separation at the anatase-rutile interfaces, reducing recombination losses. Therefore, the overall catalytic performance reflects a balance between light absorption, charge transport, and surface reaction kinetics, rather than band gap modification alone.

The results indicate that the degradation mechanism is primarily governed by a photo-assisted indirect oxidation process mediated by electrogenerated active chlorine species (HClO, Cl_2_, and ClO^−^). In the NaCl supporting electrolyte, anodic electrolysis promotes the continuous formation of these oxidants [36,56]. During irradiation (see reaction, Equation (2)), the photolysis of the accumulated active chlorine generates highly reactive radicals, such as hydroxyl (·OH) and chlorine (Cl·) radicals.


(2) HClO+hv→·OH+Cl·

This photo-activation pathway accounts for the enhanced degradation performance observed at extended reaction times. Conversely, in the Na_2_SO_4_ electrolyte, the absence of active chlorine precursors suppresses this photo-driven indirect pathway, resulting in a notably limited degradation extent [56]. The slight removal activity observed for the mixed-phase electrode in this sulfate medium can be attributed to partial direct anodic oxidation or the localized generation of weakly adsorbed hydroxyl radicals.

Although enhanced degradation under irradiation suggests a synergistic interaction between photocatalytic and electrochemical processes, direct experimental evidence such as photocurrent measurements or dark-condition controls was not obtained in this study. Therefore, the proposed synergy is inferred from comparative degradation behavior and optical characterization. Future work will focus on electrochemical and photoelectrochemical measurements to quantitatively assess the contribution of photogenerated charge carriers to the overall reaction mechanism.

The enhanced activity observed for the black TiO_2_-coated electrodes under irradiation can be associated with the modified electronic structure generated during the hydrogen reduction treatment. The introduction of oxygen vacancies and Ti^3+^ defect states creates localized electronic levels within the band structure, which improve light absorption and facilitate the generation of photogenerated charge carriers under UV-Vis irradiation. These carriers can participate in interfacial oxidation reactions and may contribute to the photo-assisted activation of electrogenerated chlorine species such as HOCl and OCl^−^ in the chloride electrolyte. As a result, the presence of black TiO_2_ promotes a synergistic interaction between photocatalytic processes and electrochemical oxidation pathways, leading to improved degradation efficiency compared with uncoated electrode.

Although the dominant degradation pathway observed in this study corresponds to indirect oxidation mediated by electrogenerated chlorine species, the possibility of a minor contribution from direct oxidation processes cannot be completely excluded. Under irradiation, photogenerated holes in the valence band of black TiO_2_ may participate in direct oxidation reactions involving acetaminophen molecules adsorbed on the electrode surface or chloride ions present in the electrolyte. However, the limited degradation observed in sulfate medium indicates that such direct pathways play a secondary role compared with the indirect oxidation mechanism driven by active chlorine species.

The stability of the defect structure generated during the hydrogen reduction treatment is also an important factor when considering the behavior of black TiO_2_ under electrochemical conditions. During anodic operation in chloride electrolyte, highly oxidative species such as HOCl and Cl_2_ are continuously produced, which may partially re-oxidize surface Ti^3+^ species or passivate oxygen vacancies at the outermost surface. Nevertheless, the bulk defect structure created during the reduction process is generally more stable and continues to influence the electronic properties and charge-transfer behavior of the material. Therefore, although some surface transformation of defect sites may occur during operation, the modified electronic structure of black TiO_2_ is expected to remain sufficiently stable to sustain its photoelectrochemical activity during the experimental timeframe investigated in this study.

Furthermore, the HPLC analysis revealed the emergence of two additional chromatographic peaks at retention times distinct from that of acetaminophen. Although these intermediates were not quantitatively assessed, their detection experimentally confirms the stepwise degradation of the target molecule, likely through aromatic ring cleavage or substitution reactions induced by the combined action of active chlorine and the photogenerated radicals [36].

An analysis of variance (ANOVA) was performed to evaluate the influence of the experimental variables on degradation performance. As shown in Table 4, the type of supporting electrolyte was identified as the dominant factor (*p* = 1.52 × 10^−7^), exceeding the influence of the precursor phase used during coating. These statistical results are consistent with experimental evidence highlighting the key role of chlorine species generation in the degradation of acetaminophen.

The stability of Ti^3+^ centers and oxygen vacancies under anodic conditions in chloride electrolyte remains an important consideration. Under highly oxidative environments, these reduced species may undergo partial re-oxidation or participate in side reactions with active chlorine species. Although no post-reaction surface characterization (e.g., XPS) was conducted in this study, the potential evolution of defect states during operation cannot be excluded. This aspect will be addressed in future work to evaluate long-term stability and its impact on catalytic performance.

However, the precursor type also showed a statistically significant effect (*p* = 9.65 × 10^−3^), indicating that the precursor selected for the synthesis of black TiO_2_ contributes to measurable differences in degradation performance due to its distinct physicochemical properties. The differences in degradation efficiency among precursor phases can also be correlated with the defect structures inferred from PL analysis. Anatase-based samples exhibited stronger defect-related emission, indicating a higher density of localized states that may promote charge carrier recombination. In contrast, rutile and P25 samples showed a redistribution or attenuation of these emissions, suggesting more efficient charge separation or reduced recombination rates. In the case of P25, the coexistence of anatase and rutile phases may further facilitate carrier separation across interfaces. These variations in defect distribution and recombination dynamics provide a phase-dependent explanation for the observed electrochemical performance.

The experiments were conducted at a fixed current density; however, this parameter is expected to significantly influence the degradation mechanism. Increasing the applied current density can enhance the generation rate of active chlorine species, potentially improving degradation efficiency but also promoting competing side reactions such as oxygen evolution or excessive chlorine formation. Variations in current density may also affect the relative contribution of electrode material properties, potentially altering the observed performance ranking among precursor phases. A systematic evaluation of this parameter is therefore warranted and will be addressed in future studies.

**Figure 9:**
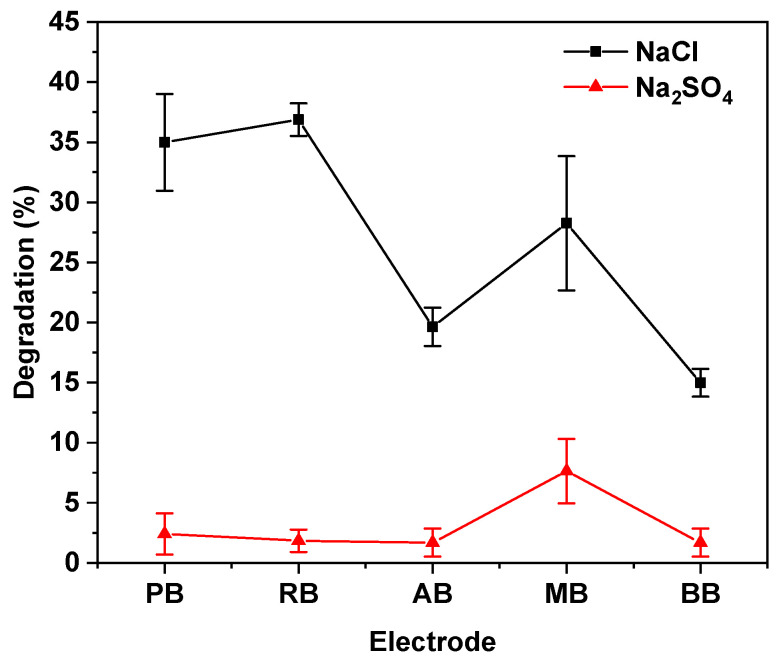
ANOVA interaction plot showing the combined effects of precursor phase and electrolyte composition on acetaminophen degradation.

Figure 9 presents the interaction plot, which confirms that the type of supporting electrolyte is the main factor governing the efficiency of the photoelectrooxidation process. For all electrode precursors, the average degradation obtained in the NaCl supporting electrolyte is higher than that observed in Na_2_SO_4_, which is consistent with the statistical significance revealed by the ANOVA results. In the Na_2_SO_4_ supporting electrolyte maintained low degradation values that are largely indistinguishable among the different coating types. Conversely, in the NaCl medium, rutile- and P25-based coated electrodes (RB and PB) exhibit the highest degradation efficiencies (45%), whereas the uncoated electrode (BB) shows the lowest response.

The higher degradation efficiency observed for rutile- and P25-derived electrodes compared with anatase-based ones may be related to intrinsic differences in the electronic and structural properties of these TiO_2_ phases. Rutile typically exhibits higher electrical conductivity and improved charge carrier mobility, which can facilitate electron transfer during electrochemical reactions occurring at the electrode surface. In the case of P25, the coexistence of anatase and rutile phases can promote interfacial charge separation, reducing the recombination of photogenerated charge carriers and improving catalytic efficiency. Additionally, variations in the density and distribution of defects generated during the hydrogen reduction treatment may influence the interaction between the electrode surface and reactive chlorine species present in the chloride electrolyte. These combined factors may contribute to the enhanced degradation performance observed for rutile- and P25-based electrodes in the present study.

The enhanced performance of the black TiO_2_ electrodes can primarily be attributed to the formation of oxygen vacancies and Ti^3+^ defect states generated during the hydrogen reduction treatment. These defects modify the electronic structure of TiO_2_ and improve charge carrier transport properties. Although the coexistence of different crystalline phases, particularly in P25-derived materials, may contribute to improved charge separation through heterojunction formation, the dominant factor influencing the photoelectrochemical behavior appears to be the defect structure introduced during the reduction process.

The relatively high performance of P25-derived electrodes may also be associated with the intrinsic mixed-phase composition of this material. P25 typically contains both anatase and rutile phases, which can form interfacial heterojunctions that facilitate charge separation and reduce the recombination of photogenerated carriers. After the hydrogen reduction treatment, the resulting black TiO_2_ is expected to retain this mixed-phase structure while incorporating defect states such as oxygen vacancies and Ti^3+^ species. This combination of structural heterojunctions and defect-induced electronic states may contribute to the enhanced catalytic performance observed for P25-based electrodes.

It should also be noted that the applied current density represents a key operational parameter in electrochemical oxidation processes. Increasing the current density generally enhances the generation rate of active chlorine species in chloride-containing electrolytes, which can accelerate pollutant degradation through indirect oxidation pathways. However, excessively high current densities may also promote competing reactions such as oxygen evolution or chlorine gas formation, which reduce current efficiency and increase energy consumption. Conversely, lower current densities may limit the production of reactive oxidants and slow the degradation kinetics. Although the present study employed a constant current density to isolate the effect of the precursor phase, future studies could explore the optimization of this parameter to further improve the performance of black TiO_2_-coated electrodes.

From an application perspective, energy consumption represents an important parameter when evaluating the feasibility of electrochemical water treatment technologies. Metrics such as the electrical energy per order (EEO) are commonly used to compare the efficiency of advanced oxidation processes. Although the present work focused primarily on understanding the influence of the TiO_2_ precursor phase on catalytic behavior, the optimization of operational parameters such as current density, electrode configuration, and electrolyte composition will be essential for minimizing energy consumption in practical applications. Future studies will therefore consider the evaluation of energy efficiency metrics to further assess the technological viability of black TiO_2_-coated electrodes.

## 4. Conclusions

Black TiO_2_ nanostructures were successfully synthesized from different crystalline precursors (P25, anatase, rutile, and a mixed phase). The hydrogen thermal treatment proved to be the key step in generating black TiO_2_, producing a consistent narrowing of the band gap in all samples and markedly enhancing visible-light absorption. This modification confirms the successful creation of oxygen vacancies and Ti^3+^ defect states, which are highly beneficial for photoelectrochemical applications. Among the studied phases, anatase exhibited the most pronounced band-gap reduction, while rutile and P25 demonstrated greater optical stability, indicating that the black TiO_2_ approach can be effectively applied to different TiO_2_ structures.

The dip-coating method enabled the fabrication of uniform and mechanically stable coatings on titanium electrodes using a six-layer configuration. The anatase-based black TiO_2_ showed the highest deposited mass, evidencing strong adhesion and favorable dispersion, which highlights its suitability for electrode fabrication. Overall, the coating process confirmed the feasibility of integrating black TiO_2_ into practical electrochemical systems.

Photoelectrooxidation experiments demonstrated that the electrolyte composition is the dominant factor controlling acetaminophen degradation. While sulfate medium resulted in negligible removal, chloride electrolyte significantly enhanced degradation, confirming the synergistic role of black TiO_2_ and electrogenerated active chlorine species (HOCl and OCl^−^). Importantly, the presence of black TiO_2_ coatings improved the overall degradation performance compared with uncoated electrodes, evidencing the catalytic contribution of the modified materials. Although anatase provided the best coating quality, rutile- and P25-derived black TiO_2_ electrodes achieved the highest pollutant removal in chloride medium, highlighting the versatility and practical potential of black TiO_2_ for photoelectrochemical water treatment.

Overall, the results demonstrate that the modification of TiO_2_ through hydrogen reduction is an effective strategy to tailor the optical and electronic properties of the material, enabling enhanced visible-light absorption through the formation of defect states. Although the improvement in acetaminophen degradation was moderate under the studied experimental conditions, the presence of black TiO_2_ coatings consistently promoted higher activity compared with the uncoated electrode. These findings indicate that the catalytic contribution of black TiO_2_ is significant but operates in conjunction with electrolyte-mediated oxidation pathways. Therefore, the combination of defect-engineered TiO_2_ materials with optimized electrochemical conditions represents a promising strategy for improving the performance of photoelectrochemical systems aimed at the removal of pharmaceutical contaminants from water.

## Figures and Tables

**Table 1:** TiO_2_ precursor phases, sample codes and compositions employed in the synthesis of black TiO_2_ materials.

Sample	ID	Composition Description
TiO_2_–P25	P25	Commercial P25 powder (mixed anatase/rutile)
TiO_2_–Rutile	R	Commercial rutile TiO_2_ powder
TiO_2_–Anatase	A	Commercial anatase TiO_2_ nanopowder
TiO_2_–Mix	M	Solid state mixture: 20% rutile/80% anatase
TiO_2_–P25 + Urea	N,C P25	P25 sample co-doped with 20% urea (solid method)
TiO_2_–Rutile + Urea	N,C R	Rutile sample co-doped with 20% urea (solid method)
TiO_2_–Anatase + Urea	N,C A	Anatase sample co-doped with 20% urea (solid method)
TiO_2_–Mix + Urea	N,C M	Mix sample co-doped with 20% urea (solid method)

**Table 2:** Direct and indirect band gap (*E_g_*) values of TiO_2_ samples before and after N,C co-doping and reductive thermal treatment, as determined by UV–Vis DRS.

Sample	Direct *E_g_* (eV)	Indirect *E_g_* (eV)
P25	3.340 ± 0.030	3.030 ± 0.027
R	3.286 ± 0.029	2.942 ± 0.026
A	3.307 ± 0.029	3.137 ± 0.028
M	3.062 ± 0.027	2.952 ± 0.026
N,C P25	3.198 ± 0.028	2.820 ± 0.025
N,C R	3.090 ± 0.027	2.856 ± 0.025
N,C A	3.259 ± 0.029	3.012 ± 0.027
N,C M	3.096 ± 0.027	2.821 ± 0.025
T–P25	3.031 ± 0.027	2.721 ± 0.024
T–R	3.014 ± 0.027	2.816 ± 0.025
T–A	2.972 ± 0.026	2.639 ± 0.023
T–M	2.987 ± 0.026	2.723 ± 0.024
T–N,C P25	2.986 ± 0.026	2.698 ± 0.024
T–N,C R	3.049 ± 0.027	2.871 ± 0.025
T–N,C A	2.970 ± 0.026	2.639 ± 0.023
T–N,C M	3.028 ± 0.027	2.705 ± 0.024

**Table 3:** Mass of black TiO_2_ deposited on anode and cathode titanium substrates for each precursor phase using the dip-coating method.

Black TiO_2_ Sample	Anode Mass (g) m ± 0.0001	Cathode Mass (g) m ± 0.0001
P25	0.0037	0.0034
R	0.0029	0.0031
A	0.0041	0.0043
M	0.0034	0.0037

**Table 4:** ANOVA results evaluating the influence of electrolyte composition and precursor phase on acetaminophen degradation efficiency during photoelectrooxidation.

Source of Variation	Sum of Squares (sum_sq)	Degrees of Freedom (df)	F-Value (F)	*p*-Value (PR(>F))
C(Electrolyte)	0.285560	1	165.37	1.52 × 10^−7^
C(Precursor)	0.041845	4	6.058	9.65 × 10^−3^
C(Precursor):C(Electrolyte)	0.035761	4	5.177	1.60 × 10^−2^
Residual	0.017268	10	---	---

## Data Availability

The data supporting the findings of this study are available from the corresponding author upon reasonable request.
